# HSV-1 UL56 protein recruits cellular NEDD4-family ubiquitin ligases to suppress CD1d expression and NKT cell function

**DOI:** 10.1128/jvi.02140-24

**Published:** 2025-03-06

**Authors:** Lingxi Qiu, Xuedi Gao, Xinyue Shao, Jingwen Xi, Siyang Chen, Thanh Pham, Yi Wang, Jonathan Dong, Samhita Divakar Rao, Jingting Hao, Jae Ho Lo, Rirong Yang, Esteban A. Engel, Colin M. Crump, Weiming Yuan

**Affiliations:** 1 Department of Molecular Microbiology and Immunology, Keck School of Medicine, University of Southern California 98523 , Los Angeles, California, USA; 2 Princeton Neuroscience Institute, Princeton University 12223 , Princeton, New Jersey, USA; 3 Department of Pathology, University of Cambridge 438829 , Cambridge, United Kingdom; University of Toronto, Toronto, Ontario, Canada

**Keywords:** UL56, CD1d, NKT, Nedd4L, immune evasion, viral pathogenesis, herpesviruses

## Abstract

Herpesviruses, including α-herpesvirus and herpes simplex virus (HSV-1), are masters of immune evasion. Previously we demonstrated that CD1d-restricted NKT cells are required for optimal anti-HSV-1 immune responses and HSV-1 efficiently downregulates CD1d to suppress NKT cell function. To delineate how the virus evades NKT cell function and establishes infection *in vivo*, we screened an HSV-1 expression library to identify the viral gene(s) downregulating CD1d and discovered that a leaky late gene, UL56, most efficiently suppresses CD1d expression by degrading the protein, apparently via both proteasome- and lysosome-dependent pathways. To investigate the molecular mechanism of UL56 suppression of CD1d expression, we purified and identified UL56-associated proteins by mass spectrometry. The most abundant associated proteins were members of NEDD4 E3 ubiquitin ligase family. Interestingly overexpression of one member, NEDD4L is sufficient to downregulate CD1d expression. However, different from the K5 protein from Kaposi sarcoma’s herpesvirus (KSHV), UL56 and NEDD4L did not directly ubiquitinate CD1d. CD1d protein lacking the key lysine residue in its cytoplasmic tail is similarly downregulated by UL56 and NEDD4L protein. Co-expression of UL56 and NEDD4L synergistically reduced the CD1d expression, suggesting that UL56 collaborates with NEDD4L to downregulate CD1d. During *in vivo* infection, UL56-deficient mutant virus showed significantly weaker virulence in NKT-sufficient mice but demonstrated higher virulence in mutant mice lacking NKT cells. All our results supported that at least one of the pathogenesis functions of UL56 is its suppression of NKT cell function during infection.

**IMPORTANCE:**

In the large DNA genomes of herpeviruses, there are many genes encoding associate proteins. Most of these proteins are not essential for viral replication but play key roles in viral pathogenesis, in particular, modulating the host immune system to allow efficient viral replication *in vivo* and latency. The HSV-1 UL56 gene is one of such genes, and its exact pathogenic roles have remain elusive. After we demonstrated the essential roles of CD1d-restricted NKT cells in anti-HSV-1 immunity during HSV-1 ocular infection (P. Rao, X. Wen, J. H. Lo, S. Kim, X. Li, et al., J Virol 92:e01490-18, 2018, https://doi.org/10.1128/jvi.01490-18), we now screened the HSV-1 expression library and identified UL56 is a key factor downregulating CD1d and suppressing NKT cell function. In this manuscript, we are reporting our molecular mechanism study of how UL56 evades CD1d antigen presentation and NKT cell function.

## INTRODUCTION

With a large double-stranded DNA genome of about 150–200 kb, herpesviruses are masters of immune evasion. From the conventional MHC class I (1, 2) and MHC class II (3) to nonconventional MHC I-like CD1d (4–6) and MR1 (7, 8) molecules, herpesviruses from all three subfamilies (α, β, γ) possess a large arsenal of viral genes to downregulate these key antigen-presenting molecules to suppress the function of both adaptive T cells and innate-like natural killer T (NKT) and mucosa-associated invariant T (MAIT) cells. These immune evasion mechanisms are crucial for herpesviruses to establish their evolutional niche in immune-competent hosts.

CD1d-restricted Natural Killer T (NKT) cells are an unconventional subset of T cells co-expressing T-cell receptor (TCR) and typical surface receptors for NK cells (9). The major population of NKT cells expresses a single TCRα chain, Vα24Jα18 in humans and Vα14Jα18 in mice, and are often called invariant NKT (iNKT) cells. Innate-like NKT cells are among the first responders in the periphery during immune responses and are typically activated within hours. Upon activation, they rapidly produce copious amount of cytokines, both Th1 and Th2 types, and play potent immunomodulatory functions for ensuing adaptive immune responses (10). Different from MHC class I and II, CD1d molecules present antigenic phospholipids or glycolipids, rather than peptide ligands, to NKT cells (11, 12). Many viruses, including HIV (13–15), HSV-1 (4, 16), KSHV (5), and LCMV (17), use different mechanisms to suppress CD1d and NKT cell function. A common tactic adopted by viruses to evade T cell function is to downregulate the antigen-presenting molecules (18, 19). In most viruses studied so far, they achieve the blocking of NKT cell function via down-regulation of CD1d expression (4, 5, 17).

Previously, we have demonstrated that CD1d-restricted NKT cells play an important role in anti-HSV-1 immunity (20). Therefore, for HSV-1 to establish successful infections, overcoming NKT cell function is necessary. We have also demonstrated that at least one mechanism used by HSV-1 to evade NKT cell function is to suppress CD1d expression in antigen-presenting cells (4, 20). For the viral evasion genes, we first discovered that the serine/threonine protein kinase, US3, rapidly phosphorylates the major motor protein, KIF3A, and downregulates CD1d expression (20, 21). Using a US3-deficient virus, we demonstrated that US3 is required for optimal CD1d downregulation during infection (20). Nevertheless, when compared to wild-type virus, US3-deficient virus still suppresses CD1d expression, suggesting that there are additional viral proteins contributing to the inhibition of CD1d expression. We, therefore, employed an expression library of HSV-1 and screened for additional viral gene(s) that contribute to CD1d suppression. Here, we reported that UL56, an adaptor protein of cellular ubiquitin ligases, is another major viral protein that participates in the modulation of CD1d expression.

## RESULTS

### UL56 is a major HSV-1 protein inhibiting CD1d antigen presentation and targeting CD1d for degradation

**Fig 1:**
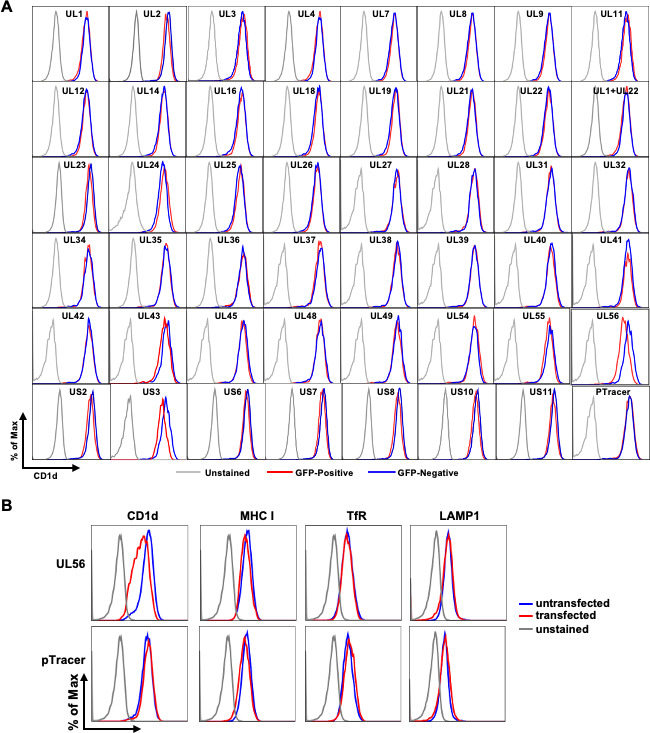
(**A**) Identification of HSV-1 UL56 protein as a potent immune evasin downregulating CD1d. 293T.CD1d cells were transfected with GFP-expressing pTracer alone or co-transfected with plasmids expressing individual HSV-1 genes from an expression library. (**B**) Specificity of UL56 downregulation of CD1d expression. 293T.CD1d cells were GFP-expressing pTracer plasmid alone or co-transfected with the pcDNA3 construct expressing HSV-1 UL56 protein. Forty-eight hours post transfection, cells were then stained for cell surface CD1d expression with mouse monoclonal antibody, CD1d.51.1 and analyzed by flow cytometry. Transfected and untransfected were identified as GFP-positive (in red) and GFP-negative (in blue) cells, respectively.

We employed an HSV-1 expression library (22) to express individual viral genes in a 293T cell line stably expressing the human CD1d gene, 293T.CD1d cells (23). Plasmids expressing individual viral genes were co-transfected with pTracer plasmid DNA. Co-expressed GFP protein was used as a surrogate marker for viral gene expression as we previously reported (21, 23). Transfected cells were stained with a mouse monoclonal anti-human CD1d antibody, CD1d.51.1. Transfected and untransfected cells were gated based on GFP expression. The cell surface expression of CD1d was compared between untransfected and transfected cells expressing individual viral genes. As expected, the expression of the serine/threonine protein kinase US3 efficiently downregulated CD1d (Fig. 1A). Among the rest of viral genes, UL56 was the major viral protein that substantially downregulated CD1d expression, while other viral proteins either had no effect or only had minor CD1d downregulation. Of note, the glycoprotein B (gB) does not downregulate on its own, as we reported previously (23). Nevertheless, many viral membrane glycoproteins have been implicated in immune evasion mechanisms (24). In addition to UL27/gB, UL1/gL, and UL22/gH in our initial screening (Fig. 1A), we have re-cloned the other glycoproteins that were not included in the screening due to low expression. Expression of these glycoproteins was verified, but none of the glycoproteins led to CD1d downregulation (Fig. S1A and B). One of the other prominent HSV-1 immune evasion proteins is US12/ICP47, which potently downregulates MHC class I molecules and suppresses CD8 T cell function (25, 26). We, therefore, expressed ICP47 using a new plasmid construct and detected no CD1d downregulation (Fig. S1B). Therefore, UL56 protein is the other major HSV-1 protein downregulating CD1d expression in addition to the viral US3 kinase (20, 21).

To investigate how conserved the UL56-mediated CD1d downregulation, we surveyed the UL56 sequences in different HSV-1 strains. The UL56 amino acid sequences are conserved between Strain KOS (NCBI, sequence ID: AFE62885.1) and Strain 17 (NCBI, sequence ID: YP_009137132.1). However, in another commonly used experimental strain F, there are two amino acid variations (T126S and W213G) in UL56 amino acid sequences (NCBI, sequence ID: ADD60011.1) (Fig. S2A). We therefore generated UL56-F construct by mutating the two amino acids and expressed it in 293T.CD1d cells. The comparable CD1d downregulation suggested that this immune evasion function is conserved among different HSV-1 strains (Fig. S2B).

To examine the specificity of UL56 downregulation of CD1d expression, we expressed UL56 protein in 293T.CD1d cells and analyzed the cell surface expression of other cell surface membrane proteins. The expression of two common cell surface markers, transferrin receptor (TfR)/CD71 and LAMP1, was not affected by UL56 expression (Fig. 1B). Importantly, LAMP1, similar to CD1d protein, is present both at cell surface and intracellularly at late endosomal/lysosomal compartments (27). We detected that another antigen-presenting molecule, MHC class I stained by the pan-anti-HLA A/B/C monoclonal antibody, W6/32, was slightly downregulated (Fig. 1B). However, expression of GFP in control transfection also led to similar level of MHC class I downregulation, suggesting the slight downregulation is non-specific and due to the transfection procedure. All these results suggested that HSV-1 UL56 specifically downregulates CD1d.

**Fig 2:**
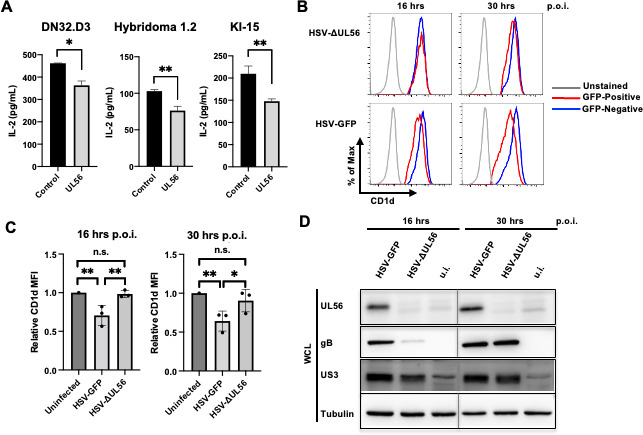
UL56 inhibits NKT cell function and is critical for optimal CD1d downregulation during HSV-1 infection. (**A**) 293T.CD1d were transfected with either pTracer (control) or pcDNA3 construct expressing UL56 (UL56). Transfected cells were either loaded with α-galactosylceramide (α-GalCer) and co-cultured with indicated iNKT cell hybridomas. Cell culture media were subjected to mouse IL-2 (mIL-2) ELISA. The average and standard deviations of IL-2 amounts were calculated from three independently repeated experiments and plotted. (**B–D**) HeLa.CD1d cells were infected by HSV-1-GFP or UL56-deficient HSV-1-GFP at m.o.i. of 1 for indicated times and subjected to staining with anti-CD1d monoclonal antibodies, CD1d-51.1 and analyzed by flow cytometry. Uninfected and infected cells were gated as GFP-negative and GFP-positive cells, respectively. (**C**) The experiments were repeated three times and relative MFI values of CD1d staining were calculated as (CD1d MFI of GFP-positive cells)/(CD1d MFI of GFP-negative cells). (**D**) HeLa.CD1d cells, uninfected (u.i.) or infected with HSV-1 viruses at m.o.i. of 1 for indicated times. Cell lysates were subjected to western blotting with indicated antibodies to examine the expression of viral proteins, UL56, glycoprotein B (gB), and US3. Cellular tubulin protein was blotted as a loading control. Statistical analysis by Student’s *t* test or one-way ANOVA with Duncan *post hoc* multiple comparisons. n.s., not significant. *P < 0.05, **P < 0.01.

To examine whether the UL56-induced CD1d downregulation has a functional impact on NKT cell function, we performed antigen presentation assays using UL56-expressing 293T.CD1d as antigen-presenting cells. The cells were loaded with the prototype iNKT cell ligand, α-galactosylceramide (α-GalCer), and co-cultured with iNKT cell hybridomas with different TCRs: DN32.D3, Hyb1.2, or KI-15. For all iNKT cell clones, expression of UL56 in antigen-presenting cells led to lower NKT cell activation measured by IL-2 secretion (Fig. 2A), suggesting that UL56 suppression of CD1d expression resulted in inhibition of NKT cell function. To ensure that the NKT cell stimulation is CD1d-dependent, we also performed the antigen presentation assay using 293T cells. Loading α-GalCer to these cells and co-culture with neither D32.D3 nor Hyb1.2 hybridoma cell line led to NKT cell activation (Fig. S3A), confirming the specificity of CD1d antigen presentation to NKT cells in 293T.CD1d cells.

To examine whether UL56 is important in downregulating CD1d expression during HSV-1 infection, we employed a recently generated UL56-deficient virus. HeLa.CD1d cells were infected at m.o.i. of 1 with either wild-type or UL56-deficient virus mutant. Both the wild-type (WT) virus (28) and the UL56-deficient virus expressed GFP protein. At m.o.i. of 1, approximately 30% of cells were infected and GFP-positive. The cells were examined at different time points post infection and subjected to flow cytometry to examine the CD1d downregulation. At 16 hours post infection, while CD1d was clearly downregulated in cells infected by wild-type HSV-1 viruses, there is no detectable CD1d downregulation in cells infected with mutant viruses (Fig. 2B and C). At 30 hours post infection, slight downregulation of CD1d can be detected in mutant virus-infected cells, while there is significantly more downregulation in WT virus-infected cells. The low-level CD1d downregulation in cells infected by UL56-deficient viruses was likely mediated by other HSV-1 genes including US3. We verified the absence of UL56 expression in mutant virus-infected cells using anti-UL56 antibodies (Fig. 2D). The rabbit anti-UL56 antibody was generated using GST-fused UL56 protein purified from *E. coli* bacteria. Interestingly, the absence of UL56 gene in mutant viruses apparently affected the expression kinetics of other viral genes, US3 and glycoprotein B (gB). For both proteins, there was low expression at 16 hours post infection, but both of them caught up by 30 hours post infection (Fig. 2D). This may explain the relatively low CD1d downregulation at 16 hours post infection by UL56-deficient viruses, compared to that in cells ectopically expressing US3 (Fig. 1).

We previously reported that HSV-1 US3 protein kinase, by phosphorylating the major cellular motor, KIF3A, blocks the CD1d outbound trafficking and suppresses the CD1d expression (21). To investigate the mechanism of UL56 downregulation of CD1d expression, we first examined the relationship between the two viral evasins during CD1d downregulation. 293T.CD1d cells were transfected with plasmid constructs expressing US3 and UL56 either independently or simultaneously. Surface CD1d expression was measured by flow cytometry. Interestingly, co-expression of the two viral proteins significantly increased the CD1d downregulation (Fig. S3B and C), suggesting that the two viral proteins employed different mechanisms and collaborated to downregulate CD1d expression.

**Fig 3:**
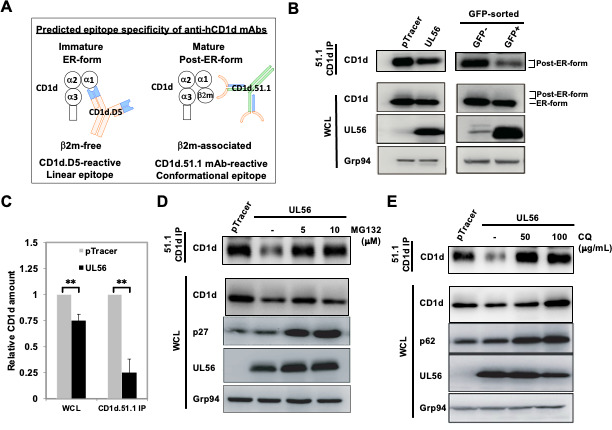
UL56 expression leads to CD1d degradation. (**A**) Predicted epitope specificity of two mouse anti-human CD1d (hCD1d) monoclonal antibodies, CD1d.D5 and CD1d.51.1. (**B–E**) 293T.CD1d were transfected with pTracer alone or together with pcDNA3 construct expressing UL56 protein (UL56), then either untreated (**B, C**) or treated with MG132 or chloroquine (CQ) at indicated concentrations (**D, E**). Upon transfection, 293T.CD1d cell lysates were subject to immunoprecipitation with anti-CD1d antibody, CD1d.51.1 and western blotting with anti-CD1d antibody, CD1d.D5. (**B**) Decrease of CD1d protein levels upon UL56 expression. Immature or mature forms of CD1d proteins were denoted as “ER-form” and “Post-ER-form”, respectively. Cellular Grp94 protein was blotted as a loading control. (**C**) Quantitation of CD1d protein levels upon UL56 expression. Relative amounts of mature CD1d protein were quantitated from immunoprecitated CD1d protein bands with Bio-Rad Imaging Lab software after chemiluminescence detection. Relative CD1d amount was calculated as (Relative amount of CD1d upon pTracer or UL56 expression)/(Relative amount of CD1d upon pTracer expression). The average and standard deviations of relative CD1d amounts were calculated from three independently repeated experiments and plotted. Statistical analysis by Student’s *t* test. n.s., not significant. **P < 0.01.

To investigate the molecular mechanism of UL56 downregulation of CD1d expression, we examined the protein levels of CD1d upon UL56 expression. CD1d protein exists in two forms in professional antigen-presenting cells and cells ectopically expressing CD1d, an immature ER-localized form and a mature post-ER-form (29–31). These two forms of CD1d can be specifically recognized by two mouse anti-human CD1d monoclonal antibodies, D5 and CD1d51.1, respectively (Fig. 3A,[29, 32]). The two antibodies specifically recognize a linear epitope in the ER-form or a conformational epitope in the post-ER-form CD1d proteins (30, 33). Due to their different glycosylation modification, ER-form CD1d proteins form a sharp and dominant band due to the uniformly high-mannose glycosylation, while post-ER form CD1d proteins form a faint smear above the ER-form CD1d band in western blots of whole cell lysate but can be enriched after immunoprecipitation by the conformation-dependent CD1d.51.1 monoclonal antibody and form a band (Fig. 3B first and second panels) (31, 34). Transient expression of UL56 protein in 293T.CD1d led to a clear decrease of mature CD1d protein as detected by western blotting of CD1d.51.1-immunoprecipitated CD1d proteins (Fig. 3B, first panel). The ER-form CD1d protein was also decreased to a lesser extent (Fig. 3B, second panel). Since in the 293T.CD1d stable cell line, CD1d is expressed under the control of CMV promoter and no regulation of transcription or translation is expected. Therefore, these results suggested that CD1d may be degraded upon UL56 expression. The efficiency of transient transfection is usually variable, typically varying from 20%–30% to 60%–70%. To more accurately evaluate the extent that UL56 led to CD1d degradation, we sorted GFP-positive cells from cells transfected with pTracer.UL56 plasmids, GFP-positive or -negative cells were compared for CD1d protein level. Remarkably, UL56 expression led to approximately degradation of 75% mature CD1d proteins, while only about 25% ER-form CD1d is degraded (Fig. 3B, right panels and Fig. 3C), suggesting that UL56 more efficiently targets the mature form of CD1d protein for degradation.

To further delineate the mechanism of UL56-mediated CD1d degradation, we employed the two specific inhibitors for proteasome and lysosomal degradation, MG132 and chloroquine, respectively. The p27Kip1 (an inhibitor protein of cyclin-dependent kinases) is degraded by the proteasome (35), while the p62 protein (sequestosome 1, SQSTM1) is a ubiquitin-binding scaffold protein degraded by lysosomes. After treatment by MG132 or chloroquine, substantial accumulation of p27 and p62 proteins was detected, respectively, confirming the inhibition of proteasomal or lysosomal function. Interestingly, both inhibitors rescued CD1d from degradation (Fig. 3D and E) suggesting that both degradation pathways may be involved in UL56-mediated degradation of CD1d.

### UL56 collaborates with cellular NEDD4 family E3 ligases to induce CD1d degradation

**Fig 4:**
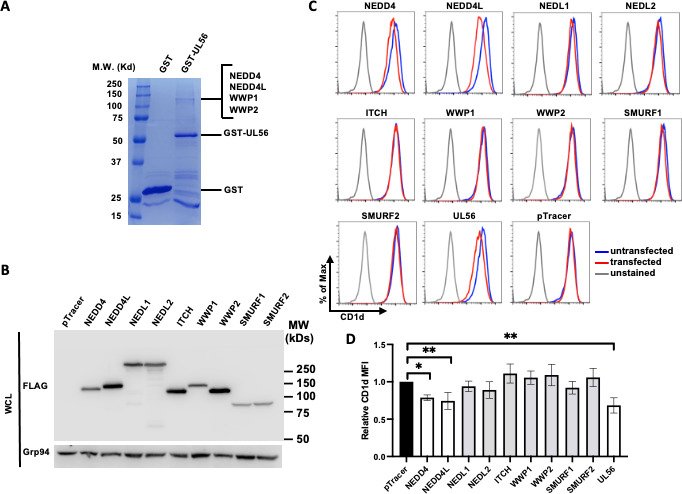
UL56 interacts with NEDD4-family E3 ubiquitin ligases and may recruit to suppress CD1d expression. (**A**) Cellular proteins associated with HSV-1 UL56 protein were purified from cell lysates of transfected 293T.CD1d cells expressing GST-fused UL56 (1–215) by GST-pulldown. The high-molecular-weight proteins co-purified with GST-UL56 protein were subjected to mass spectrometry analysis to identify these proteins. Cells expressing GST protein were used as a control. (**B–D**) Individual members of FLAG-tagged NEDD4-family E3 ubiquitin ligases were co-transfected with pTracer plasmid in 293T.CD1d cells. Transfected cells were subjected to western blotting to verify the expression of individual E3 ubiquitin ligases (**B**) or stained with anti-CD1d antibody, CD1d.51.1 and cell surface CD1d expression was analyzed by flow cytometry (**C, D**). Transfected and untransfected cells were gated as GFP-positive and GFP-negative cells, respectively. (**D**) Quantitation of CD1d downregulation upon expression of NEDD4-family E3 ubiquitin ligases. The relative CD1d MFI was calculated as (CD1d MFI of CD1d in GFP-positive cells)/(CD1d MFI in GFP-negative cells). The average and standard deviations of relative CD1d MFI were calculated from three independently repeated experiments and plotted. Statistical analysis by one-way ANOVA with Duncan *post hoc* multiple comparisons. n.s., not significant. *P < 0.05, **P < 0.01.

To delineate the molecular mechanism of UL56-led CD1d degradation, we performed a GST-pull down assay to identify the cellular proteins associated with UL56 protein. HSV-1 UL56 protein is a C-terminal-anchored type II transmembrane protein with a cytosolic ectodomain (36–38). To facilitate the purification of UL56-associated proteins, we fused UL56 ectodomain (amino acids 1–215) to GST protein. Either GST or GST-fused UL56 ectodomain was expressed in 293T cells, and cell lysates were subjected to pull-down assays with glutathione beads. After extensive washes, the associated proteins were eluted, resolved on an SDS-PAGE gel, and stained by Coomassie blue. There were several protein bands present in GST-UL56 (1-215) pull-down sample, but absent in GST pull-down sample suggesting these proteins interact with the UL56 ectodomain (Fig. 4A). These protein bands were subjected to tandem mass spectrometry (MS/MS) for protein identification. Peptides from NEDD4, NEDD4L, WWP1, and WWP2 proteins were detected in the major band, while other bands contained peptides from another member in the NEDD4 E3 ligase family, ITCH. These results were consistent with earlier yeast two-hybrid screens with UL56 from herpes simplex virus-2 (39, 40) as well as a more recent report demonstrating co-precipitation of UL56 protein with multiple NEDD4 E3 ligase family members, including NEDD4, NEDD4L, WWP1, WWP2, and ITCH (41). To examine whether these E3 ligases are involved in HSV-1 UL56-mediated CD1d degradation, we first individually expressed all nine members of NEDD4 E3 ligase family. The expression of FLAG-tagged individual E3 ligase members was verified by western blotting (Fig. 4B). Interestingly, expression of NEDD4L or NEDD4 alone is sufficient to significantly downregulate CD1d expression, with NEDD4L being more efficient (Fig. 4C and D).

**Fig 5:**
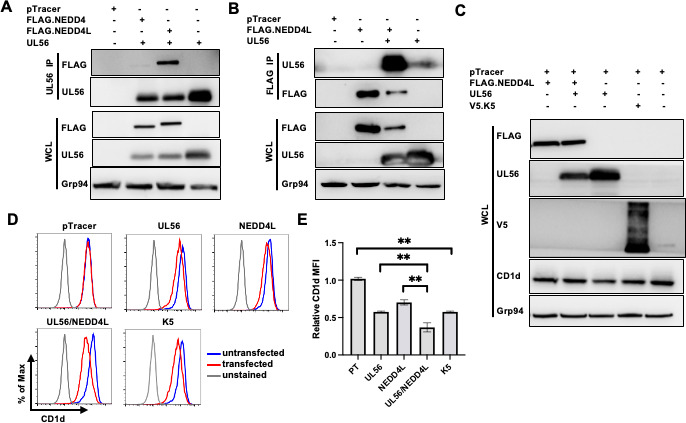
NEDD4L is the major NEDD4-family ubiquitin ligase interacting with UL56 and cooperating with UL56 to suppress CD1d expression. (**A, B**) 293T.CD1d cells were transfected with plasmids expressing UL56 alone or together with FLAG-tagged NEDD4L and subjected to co-immunoprecipitation with anti-UL56 antibodies or anti-FLAG antibodies. Immunoprecipitates were western blotted with anti-FLAG or anti-UL56 antibodies, respectively. Cellular Grp94 protein was used as a loading control. (**C–E**) 293T.CD1d were transfected with either pTracer alone or together with plasmids to express UL56 alone, NEDD4L alone, UL56 plus NEDD4L, or KSHV K5 proteins and subjected to western blotting (**C**) or cell surface staining for CD1d expression (**D**). Transfected and untransfected cells were gated as GFP-positive and GFP-negative cells, respectively. (**E**) Quantitation of CD1d downregulation. The relative CD1d MFI was calculated as (CD1d MFI of CD1d in GFP-positive cells)/(CD1d MFI in GFP-negative cells). The average and standard deviations of relative CD1d MFI were calculated from three independently repeated experiments and plotted. The experiments were repeated three times and results were summarized. Statistical analysis by one-way ANOVA with Duncan *post hoc* multiple comparisons. n.s., not significant. *P < 0.05, **P < 0.01.

To test the hypothesis that UL56 collaborates with NEDD4/4L to downregulate CD1d expression, we first performed co-immunoprecipitation assays to examine their potential interaction. Plasmids expressing UL56 and NEDD4 or NEDD4L were transfected to 293T.CD1d cells, either alone or in combination and cell lysates were subjected to co-immunoprecipation using anti-UL56 antibodies. We detected a stronger interaction between UL56 and NEDD4L, compared to that of UL56 with NEDD4 proteins (Fig. 5A). Interestingly, UL56 is in lower amounts when co-expressed with NEDD4 or NEDD4L, suggesting that these NEDD4 E3 ligases can lead to degradation of this adaptor protein, UL56. Reciprocal co-precipitation of NEDD4L proteins also efficiently co-purified with UL56 proteins (Fig. 5B). The remainder of the experiments in this study we focused on the NEDD4L ligase, which demonstrated the most efficiency in downregulating CD1d. Importantly, when UL56 and NEDD4L protein were expressed together, we could detect a significantly higher degree of CD1d downregulation (Fig. 5C through E), suggesting that UL56 collaborates with NEDD4L in downregulating CD1d. Previously another herpesviral protein, K5 from Kaposi sarcoma herpesvirus (KSHV), has been reported to downregulate CD1d (5, 42). We included the K5 protein in this experiment as a positive control. Indeed, ectopic expression of K5 protein led to CD1d downregulation (Fig. 5C through E).

To investigate whether UL56 protein indeed interacts with endogenous NEDD4L protein, we performed co-immunoprecipitation of UL56 or NEDD4L proteins in HeLa.CD1d cells infected with HSV-1 KOS strain at m.o.i. of 5 for 24 hours. In both immunoprecipitations, NEDD4L or UL56 was co-immunoprecipitated with antibodies against their counterpart, but not with normal rabbit serum (Fig. S3D), strongly suggesting that these two proteins interact with each other in HSV-1-infected cells.

### UL56 mediates CD1d downregulation without direct ubiquitination of CD1d

**Fig 6:**
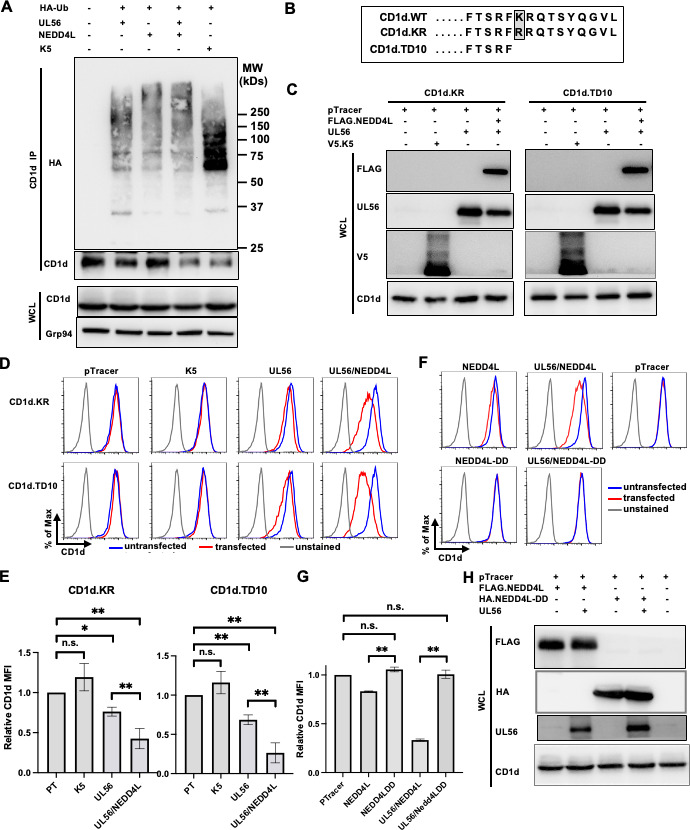
UL56 recruits NEDD4L to degrade CD1d but not via direct ubiquitination of CD1d. (**A**) 293T.CD1d cells were transfected with plasmids expressing HA-tagged ubiquitin (HA-Ub), UL56, NEDD4L or UL56 plus NEDD4L, or KSHV K5 protein. CD1d proteins were immunoprecipitated with anti-CD1d antibody, CD1d-51.1 and subjected to western blotting with HRP-conjugated anti-HA antibodies. Cellular Grp94 protein was used as a loading control. (**B–D**) 293T cells stably expressing wild-type, KR mutant or TD10 deletion mutant CD1d proteins were transfected with pTracer plasmid alone or together with plasmids expressing UL56, UL56 plus NEDD4L, or KSHV K5 proteins. Transfected cells were subjected to western blotting to verify the expression of CD1d, UL56, NEDD4L, and K5 proteins (**C**). Then, the transfected cells were stained for CD1d expression with anti-CD1d antibody, CD1d-51.1 and analyzed by flow cytometry (**D**). Transfected and untransfected cells were gated as GFP-positive and GFP-negative cells, respectively. (**E**) Quantitation of CD1d downregulation upon mutagenesis of the putative ubiquitination site in C-terminus of CD1d protein. The relative CD1d MFI was calculated as (CD1d MFI of CD1d in GFP-positive cells)/(CD1d MFI in GFP-negative cells). The average and standard deviations of relative CD1d MFI were calculated from three independently repeated experiments and plotted. (**F–H**) 293T.CD1d cells were transfected with pTracer plasmid alone or together with plasmids expressing UL56, wild-type or catalytically inactive (DD) mutant NEDD4L, or UL56 plus wild-type or mutant NEDD4L. (**F**) Transfected cells were subjected to flow cytometry after CD1d staining. (**G**) Relative CD1d expression was calculated from three replica experiments and plotted. (**H**) Western blotting was performed to verify the protein expression. Statistical analysis by one-way ANOVA with Duncan *post hoc* multiple comparisons. n.s., not significant. *P < 0.05, **P < 0.01.

Since UL56 has been reported to be an adaptor protein for NEDD4 E3 ligase family (39, 40), we hypothesized that UL56 collaborates with NEDD4L to directly ubiquitinate CD1d, similar to KSHV K5 ubiquitination of CD1d (5). To test this hypothesis, we expressed UL56, in the absence or presence of NEDD4L, with HA-tagged ubiquitin. Then, the cell lysates were subjected to monoclonal antibody CD1d.51.1 to purify mature CD1d. To our surprise, the co-expression of UL56 and NEDD4L did not lead to any detectable CD1d ubiquitination (Fig. 6A). On the other hand, upon expression of KSHV K5 protein, there are discrete bands of ubiquitinated protein bands in anti-CD1d immunoprecipitates, consistent with a previous report of K5-dependent CD1d uniquitination (5). These results suggested that UL56-mediated CD1d degradation is indirect and not via direct ubiquitination of CD1d. Rather, it is likely that expression of UL56 and associated NEDD4L leads to the degradation of a key factor that is required for the stability of mature CD1d proteins.

To test this, we generated CD1d mutants that lacked ubquitination sites. Human CD1d is a type I transmembrane protein with a short cytoplasmic tail of 13 amino acids, containing one single lysine residue (lysine 326). We generated CD1d mutants with either the single lysine (lysine 326) mutated to arginine (CD1d.KR) or with the last 10 amino acids (including the single lysine) deleted (CD1d.TD10). Stable cell lines expressing these mutant CD1d proteins were generated by retrovirally transducing 293T cells (Fig. 6B and C). These cell lines were used for ectopically expressing of UL56 with or without NEDD4L. Remarkably, both CD1d.KR and CD1d.TD10 proteins were still downregulated by UL56 protein, while K5-mediated downregulation of CD1d was completely abolished when KR and TD10 mutations were introduced (Fig. 6D and 6E). These results suggested that downregulation of CD1d by UL56 is not due to ubiquitination on its cytoplasmic tail.

To investigate whether the E3 ligase enzymatic activity is required for CD1d downregulation, we employed a catalytically inactive form of NEDD4L ligase, NEDD4L-DD, which has a C962A mutation in the HECT domain that renders the ligase catalytically inactive (43, 44). Ectopic expression of this mutant NEDD4L did not lead to CD1d downregulation (Fig. 6F lower panel and Fig. 6G), compared to that in cells expressing wild-type NEDD4L proteins (Fig. 6F upper panel, Fig. 6G and 4C). The expression of both wild-type and catalytically inactive NEDD4L as well as the co-expressed UL56 proteins was verified by western blotting in Fig. 6H. Remarkably, when the catalytically inactive form of NEDD4L ligase was co-expressed with UL56, there was complete ablation of CD1d downregulation including the CD1d downregulation mediated by UL56 itself (Fig. 6F, lower panel). These results suggested that this catalytically inactive NEDD4L mutant served as a dominant negative mutant and blocked the interaction of UL56 with endogenous NEDD4 E3 ligases for CD1d downregulation. All these results were consistent with our hypothesis that UL56 collaborates with NEDD4L protein to downregulate CD1d expression.

### UL56 is an important pathogenesis factor for *in vivo* HSV-1 infection at least partially via suppressing NKT cell function

**Fig 7:**
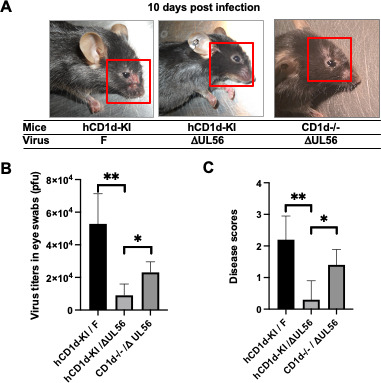
HSV-1 UL56 is required for evading NKT cell function during *in vivo* infection. hCD1d-KI or CD1d−/− mice (*n* = 5) were infected with wild-type F strain or UL56-deficient virus (ΔUL56) (5 million pfu/mouse) via cornea infection. The mice were followed up for development of eye disease (keratitis and blepharitis). Representative eye images of infected mice were presented, and periocular areas were highlighted in red boxes to show the inflammation responses upon infection (**A**). The infected mouse eyes were swabbed 2 days post infection and eye swabs were subjected to HSV-1 plaque assay in Vero cells (**B**). The severity of mouse eye infections was measured by disease scoring at 10 days post infection as in Materials and Methods (**C**). The average and standard deviations of HSV-1 titers (**B**) and disease scores (**C**) were calculated from each mouse group and plotted. Statistical analysis by one-way ANOVA with Duncan *post hoc* multiple comparisons. n.s., not significant. *P < 0.05, **P < 0.01.

To evaluate the roles of UL56 during *in vivo* HSV-1 infection, we employed an eye infection model we recently established (20) and the UL56-deficient virus we generated. Our previous studies demonstrated that HSV-1, as a human-tropic pathogen, specifically targets human, but not mouse, CD1d antigen presentation pathway for immune evasion. In order to recapitulate the HSV-1 evasion of CD1d function and more faithfully model *in vivo* HSV-1 pathogenesis, we previously generated a human CD1d-knock in (hCD1d-KI) mouse strain (45). In hCD1d-KI mice, HSV-1 could establish successful ocular infections in C57BL/6 mice, which are otherwise resistant to most laboratory strains of HSV-1 (20). Eight- to 10-week-old hCD1d-KI or CD1d-knockout (CD1d−/−) mice were inoculated with either wild-type F strain or UL56-deficient HSV-1 viruses at the dosage of 1 × 10e5 pfu/mouse. While the wild-type viruses caused clear blepharitis, severe inflammation, and severe hair loss around infected eyes, the UL56-deficient virus did not cause obvious inflammation or other symptoms in hCD1d-KI mice (Fig. 7A, left and middle panels). Importantly, in CD1d−/− mice, which lack all NKT cells (46), UL56-deficient HSV-1 infection did cause clear inflammation and mild blepharitis (Fig. 7A, right channel), suggesting that the NKT cells in hCD1d-KI mice efficiently suppressed the replication and pathogenesis of UL56-deficient virus. The examination of viral titers at 2 days post-infection also showed that the WT virus replicated to the high level, while there was little virus titer in mice infected with UL56-deficient viruses (Fig. 7B). The measured viral titers at this time point are likely to be controlled by at least two factors, the antiviral effect from the host immunity including NKT cells and the viral replication capacity of different virus strains. *In vitro*, we did not detect a substantial difference in replication capacity or plaque sizes between wild-type versus UL56-deficient viruses in Vero cells (data not shown). This is consistent with a previous report on an HSV-2 186 strain in Vero cells (47) and additional reports on different HSV-1 strains (KOS and SC16) in HaCaT keratinocytes and HFF fibroblast cells (41, 48). In CD1d−/− mice infected with the UL56-deficient virus, there were clearly more viral replication and more skin diseases compared to that in hCD1d-KI mice (Fig. 7B and C). It is unlikely that the same UL56-deficient virus has different replication capacity at single cell level in the primary target cells including keratinocytes and neuronal cells from CD1d−/− (NKT cell-deficient) or hCD1d-KI (NKT cell-sufficient) mice. These results suggested that the NKT cells in hCD1d-KI mice actively suppressed the viral replication of UL56-deficient viruses, and it is possible that the UL56 protein in wild-type HSV-1 viruses at least partially help viruses evade NKT cell function in hCD1d-KI mice to allow more robust viral replication and pathogenesis.

To investigate whether the putative NKT cell-targeting pathogenesis function of UL56 protein *in vivo* is strain-specific, we employed the UL56-null mutant generated in HSV-1 KOS strain background (41) for our ocular infection model. Eight- to 10-week-old hCD1d-KI or CD1d-knockout (CD1d−/−) mice were inoculated with either wild-type KOS strain or UL56-deficient HSV-1 viruses at the dosage of 5 × 10e6 pfu/mouse. As reported previously, KOS strain is relatively less virulent in mouse infections compared to other HSV-1 laboratory strains (49). Even at higher inoculum than that used in F strain infection (Fig. 7), wild-type HSV-1 KOS strain caused mild symptom. In most hCD1d-KI mice, there was barely any hair loss and only mild to moderate blepharitis (Fig. S2C). For UL56-deficient virus, overall, we observed milder symptoms than that of wild-type KOS strain virus, but the same UL56-deficient virus caused slightly more symptoms in CD1d−/− mice (Fig. S2C). The symptoms are too moderate to show a significant difference in disease severity among different mouse groups (Fig. S2D). Nevertheless, a distinct difference in viral titers from eye swabs was detected at 2 days post infection among different infection groups, in the same hCD1d-KI mice infected by wild-type versus UL56-deficient viruses and in hCD1d-KI versus CD1d−/− mice infected by the same UL56-deficient viruses (Fig. S2E). These results supported our hypothesis that the UL56 protein expressed in wild-type KOS strain virus helped the virus to evade the NKT cell function during its infection of hCD1d-KI mice and enhanced the viral virulence. Together with the results of HSV-1 F strain virus (Fig. 7), our studies suggested that this immune evasion mechanism is conserved among different viral strains and may represent an important virulence factor.

One caveat of our results using UL56-deficient viruses is that unwanted mutation(s) may be generated during mutagenesis and genomic recombination processes. There is a possibility that such lesion(s) in the mutant viral genomes may account for the reduced virulence or pathogenicity. It will be important in the future to generate revertant viruses with UL56 deletion repaired in the UL56-deficient viruses we have used and re-examine the phenotypes of these revertant viruses. To directly address the question, we employed a recently generated UL56-deficient virus and its revertant in HSV-1 SC16 strain background (48) and compared the viral pathogenicity of these viruses to that of wild-type SC16 strain virus. SC16 strain is another commonly used HSV-1 strain and is believed to have higher virulence than that of F and KOS strains (50, 51). We confirmed that the wild-type SC16 virus (1 × 10e5 pfu/mouse) caused severe symptoms and hair/skin loss, with a typical disease score of 3–4 in our ocular infection model (Fig. S2F and G). In fact, three of five mice died within 10 days post infection. Also, the eye diseases progressed faster in mice infected with SC16 strain HSV-1 compared to mice infected with F and KOS strains. Therefore, we surveyed the disease severity at 7 days post infection and swabbed the mouse eyes 1 day post infection as mouse eyes tended to close by 2 days post infection due to severe blepharitis and keratitis. The ocular disease was substantially milder in mice infected UL56-deficient virus or its revertant. Importantly, revertant viruses did cause more severe diseases than UL56-deficient viruses did (Fig. S2G). Also, the virus titers were higher in eyes of mice infected with revertant viruses than that in UL56-deficient virus-infected mice (Fig. S2H). All these results supported our hypothesis that HSV-1 UL56 protein is an important virulence and pathogenicity factor during ocular infections.

## DISCUSSION

### UL56 is an important viral pathogenesis factor

Our studies suggested that HSV-1 UL56 gene is a major viral protein involved in downregulating CD1d expression and suppressing NKT cell function during infection. Similar to other herpesviruses, HSV viruses contain a conserved PPXY-containing and NEDD4-interacting protein UL56, similar to ORF0 of varicella-zoster virus, UL42 of human cytomegalovirus and U24 of human herpesvirus 6A, 6B, and 7 (39, 52). Interestingly all these proteins are tail-anchored type II transmembrane proteins, suggesting they play homologous function during viral pathogenesis. Although HSV-1 contains at least 8 PPXY-containing proteins (ICP0, VP5, VP1/2, gC, VP16, UL52, UL56, and US8A) (53), UL56 contains 3 PPXY motifs and is the only one that has been shown to interact with NEDD4-family E3 ligases, as shown here and previously (39). UL56 is not essential for viral replication *in vitro* but has been demonstrated to play an important role in viral pathogenesis in vivo (41, 54, 55). Our studies suggested that part of the pathogenic role of UL56 *in vivo* is its capacity to evade NKT cell function via its downregulation of CD1d expression. In CD1d−/− mice lacking all NKT cells, mutant viruses lacking UL56 gene replicated to a higher level and the virus caused more severe diseases (Fig. 7; Fig. S2C through E). This increased virulence is unlikely due to the potential function of UL56 in viral packaging and envelopment as a candidate late domain gene (53), as these same mutant viruses replicate to higher titers than that in NKT cell-sufficient hCD1d-KI mice. It will be very interesting to further investigate whether during *in vivo* infection, the suppression of NKT cell function by UL56 collaboarates with that of US3, given that we demonstrated that these two viral proteins collaborate with each other to efficiently downregulate CD1d expression (Fig. S3B). Nevertheless, the NKT cell function in anti-HSV-1 immunity demonstrated here and our previous studies (20) supported the idea of boosting NKT cell function as a novel approach to improve the efficacies of anti-HSV-1 treatment and vaccines in the future.

### UL56 collaborates with NEDD4L to downregulate CD1d expression

Our studies strongly suggested that HSV-1 UL56, via recruiting endogenous NEDD4-family E3 ligases, particularly NEDD4L, efficiently downregulates CD1d expression in antigen-presenting cells and suppresses NKT cell function. Different members of NEDD4 gene family have been reported to regulate cell surface expression of membrane proteins, including different immune receptors (56), cell adhesion molecules (57), transporter proteins, and ion channels (58–60). In all these regulations, the substrates of the NEDD4L E3 ligases are polyubiquitinated and degraded, leading to reduced cell surface expression of the target membrane proteins. On the other hand, our result demonstrated that NEDD4L, either by itself or recruited by UL56 protein (Fig. 5D and 6A), does not downregulate CD1d by direct ubiquitination. Deletion of the only lysine residue or the C-terminal domain of CD1d protein does not impede the UL56 or NEDD4L-dependent downregulation (Fig. 6B through D). The absence of poly-ubiquitinated CD1d upon expression of UL56 and NEDD4L proteins is consistent with the results of CD1d mutagenesis (Fig. 6A). Recently, an alternative mechanism has been proposed for some ubiquitin E3 ligases to regulate protein degradation (61, 62). For example, a HECT-type E3 ligase, HACE1 functions as an adaptor protein and directly mediates the interaction between one of its substrates, Spindlin-1 and 20S proteasome subunit, which leads to the degradation of the substrate (61). It will be interesting to investigate in future studies whether NEDD4L E3 ligase mediates CD1d degradation via a similar mechanism. Another alternative mechanism for UL56-mediated CD1d degradation could be that UL56 itself or more likely, by recruiting the associated NEDD4 family E3 ligases, degrades a key protein required for CD1d stability, such as a protein required for normal CD1d maturation, its transport to cell surface, endocytosis, or recycling processes. In this scenario, the synergistic effect of CD1d downregulation by UL56 and NEDD4L proteins can be explained. UL56, as an adaptor protein, efficiently brings this protein factor to overexpressed NEDD4L protein for more effective degradation. Alternatively, overexpressed UL56 can recruit endogenous NEDD4L and other NEDD4 E3 ligase family members including NEDD4 for degrading this unknown protein factor, while the overexpressed NEDD4L protein can cooperate with endogenous adaptor protein(s) to degrade this protein factor. In this regard, we examined the protein level of B2M, an essential protein factor for the expression of MHC class I and MHC class I-like CD1d proteins (9, 63). Loss of B2M has been discovered to be a major factor for cancer immune evasion via downregulating MHC class I expression (63). However, overexpression of UL56 protein did not lead to significant loss of B2M(Fig. S3E).

In searching for the key factor mediating the UL56-induced CD1d downregulation, it is particularly interesting to investigate whether the cellular trafficking factor Golgi-associated PDZ and coiled-coil motif-containing protein (GOPC), the recently identified target protein of UL56-mediated degradation (41), is such a key factor. However, our preliminary results showed that knocking-down GOPC does not affect CD1d expression (Fig. S3F and G). These results suggested that different protein factor(s) other than GOPC mediated the UL56-induced CD1d downregulation. It will be very intriguing to identify such a key protein factor essential for CD1d expression.

As the key antigen-presenting molecule for potent NKT cells, CD1d supports the thymic development and peripheral activation of NKT cells. Therefore, cellular genes and factors regulating CD1d trafficking and expression in antigen-presenting cells will be critical for NKT cell development and function. NEDD4-family E3 ligases are the first cellular ubiquitin E3 ligases we discovered that can regulate CD1d expression levels. It is very important to further delineate the molecular mechanism how these E3 ligases precisely regulate the CD1d expression.

## MATERIALS AND METHODS

### Mice, viruses, cells, antibodies, and DNA constructs

Human CD1d-knock in (hCD1d-KI) mice were generated in our laboratory (45) and bred locally. CD1d-knock out (CD1d−/−) mice, which lack both CD1d1 and CD1d2 genes, were generously provided by Dr. Chyung-Ru Wang (Northwestern University). Wild-type HSV-1 KOS and F strain viruses were generous gifts from Dr. David Knipe (Harvard Medical School) or Dr. Bernard Roizman (University of Chicago, Chicago, IL) and Dr. David C. Johnson (Oregon Health and Science University), respectively. The UL56-deficient (ΔUL56) HSV-1 virus was generated in F strain background by homologous recombination and replacing the entire UL56 coding region with eGFP coding region from peGFP-C1 vector (Clontech) and recombinant mutants were selected by eGFP fluorescence. The transcription of eGFP in the ΔUL56 null mutant is driven by the native UL56 promoter. The generation of BAC clone-based UL56-deficient virus in HSV-1 KOS and SC16 strain background has been previously described (41, 48).

Two mouse monoclonal antibodies, D5 and 51.1.3, recognizing the ER-form and post-ER form of human CD1d protein, respectively, and mouse monoclonal anti-V5 (SV5-Pk1) antibody were purchased from Thermo Fisher Inc and used as instructed. Mouse monoclonal antibodies against human HLA-A,B,C (W6/32) and LAMP1 (H4A3) (BD Biosciences), β-tubulin, FLAG tag (M2) and HA tag (12CA5) and peroxidase-conjugated anti-HA (HA-7) antibody (Sigma), Rabbit antibodies against GOPC (Cell Signaling), NEDD4L (Proteintech) and β2 m (DAKO), and rat monoclonal antibodies against Grp94 (Enzo) were purchased and used as instructed. To generate a rabbit polyclonal antibody against HSV-1 UL56 protein, the soluble domain of HSV-1 UL56 (amino acids 1–215) was cloned into pGEX-2T vector (Clontech) by PCR from the pcDNA3.1.UL56 construct (22). GST-fused UL56 protein was purified by glutathione-beads (GE Healthcare) and used for rabbit immunizations (Convence).

The HeLa.CD1d and 293T.CD1d cell lines have been described previously (23). For generation of 293T cell lines stably expressing mutant CD1d proteins lacking C-terminal 10 amino acids (TD10) or with lysine 326 mutated to arginine (KR), PCR-based mutagenesis was performed and mutant CD1d genes were cloned into pLPCX (Clontech). Retroviruses were generated and transduced into 293T cells. Puromycin was used to select cells stably expressing mutant CD1d proteins as described previously (23). Mouse NKT cell hybridoma clone, KI-15, was generated from human CD1d-knock in (hCD1d-KI) mice (45) and generously provided by Dr. Steven Porcelli at Albert Einstein College of Medicine, Bronx, NY. The mouse iNKT cell hybridomas, DN32.D3, and Hyb1.2 were generously provided by Dr. Albert Bendelac (University of Chicago) and Dr. Mitchell Kronenberg (La Jolla Institute for Immunology), respectively.

The plasmid constructs expressing NEDD4 ubiquitin ligase family protein constructs expressed in the pCIneo vector (Promega) were generously provided by Dr. Wesley Sundquist at University of Utah and reported previously (64). A near-complete HSV-1 expression library was generally provided by Dr. David M. Knipe at Harvard Medical School (22). pCI vector expressing HA-NEDD4L-DD and HA-Ub were gifts from Dr. Joan Massague (Addgene plasmid # 27001; https://www.addgene.org/27001/; RRID:Addgene_27001) (43) and Dr. Linda Hicke (Addgene plasmid # 32169; https://www.addgene.org/32169/; RRID:Addgene_32169), respectively.

### Purification of UL56-associated proteins and mass spectrometry analysis

The ectodomain of HSV-1 UL56 (amino acids 1–215) was cloned by PCR from the pcDNA3.1.UL56 construct (22) into the BamHI/NotI sites pEBG vector [a gift from Dr. David Baltimore, Addgene plasmid # 22227; https://www.addgene.org/22227/; RRID:Addgene_22227 (65)]. 293T cells transiently transfected with pEBG or pEBG.UL56 plasmids were subjected to lysis in 1% CHAPS (Sigma) in TBS plus proteinase inhibitors. GST-pulldown of UL56-associated proteins were separately by multi-dimensional liquid chromatography coupled with tandem mass spectrometry (MS/MS) at the Taplin Biological Mass Spectrometry Facility, Harvard Medical School (Boston, MA). The MS/MS spectra were run against a sequence database by the program SEQUEST and associated software packages for the identification of the proteins as previously reported (21).

### Mouse ocular infection by HSV-1

Eight- to 10-week-old mice were infected via cornea as previously described (66, 67). Briefly, 8- to 10-week-old hCD1d-KI mice were anesthetized and the mouse corneas were scarified with syringe needles by gently scratching. HSV-1 virus inoculum was then added on top of the cornea and mouse eyelids were then rubbed to allow the virus absorption. From day 1 to day 3, infected mice were anesthetized and viruses in mouse eyes were swabbed with Q-tips. Absorbed viruses were then diluted and plaque-assayed on Vero cells.

### Periocular skin disease scoring

Post HSV-1 infection, the mouse periocular skin diseases were scored according to previously reported (66, 68). In this scoring system, normal or disease free was scored as 0. Only eye lid affected (blepharitis) with swelling and inflammation symptoms is scored as 1. Hair loss less than 3 mm from mouse eyes is scored as 2. Hair loss up to 50%, 50%–75%, or more than 75% of the infected side of the mouse face is scored as 3, 4, and 5, respectively. In addition, skin breakdown as indicated by bleeding and/or scabbing/swelling of the eyelid to the point of eye closure adds an additional 0.5 score.

### Procedures and statistical analysis

Western blotting, immunoprecipitation, *in vitro* antigen presentation and NKT cell activation assay, herpes simplex virus-1 infection of cultured cells, plaque assay, shRNA knockdown, flow cytometry and statistical tests were performed as previously described (4, 21, 23). The oligoIDs for two shRNA constructs targeting human GOPC gene are V2LHS_38464 (D3) and V2LHS_245620 (D6), respectively (Open Biosystems). For statistical tests, various statistical methods including Student’s *t*-test and one-way analysis of variance (ANNOVA) test were employed as indicated to analyze the data collected. Means with error bars representing standard deviation (SD) were presented. Significant differences were determined using one-way ANOVA with Duncan’s post-hoc test, *P < 0.05, **P < 0.01. All statistical analyses were performed using SPSS V29.0 software (SPSS IBM Corp).

## SUPPLEMENTAL MATERIAL

Figure S1 jvi.02140-24-s0001.tiff Examination of potential CD1d downregulation by HSV-1 glycoproteins and ICP47.

Figure S2 jvi.02140-24-s0002.tiff CD1d downregulation and evasion of NKT cell function is conserved in HSV-1 KOS and F strains.

Figure S3 jvi.02140-24-s0003.tiff Specificity and mechanism of HSV-1 UL56 downregulation of CD1d expression.

Supplemental legends jvi.02140-24-s0004.pdf Legends for Fig. S1 to S3.

ASM does not own the copyrights to Supplemental Material that may be linked to, or accessed through, an article. The authors have granted ASM a non-exclusive, world-wide license to publish the Supplemental Material files. Please contact the corresponding author directly for reuse.
