# Supplementary figures and images for: HSV-1 UL56 protein recruits cellular NEDD4-family ubiquitin ligases to suppress CD1d expression and NKT cell function
Source: J Virol. 2025 Mar 6;99(4):e02140-24. doi: 10.1128/jvi.02140-24 (PMC11998485; doi:10.1128/jvi.02140-24)

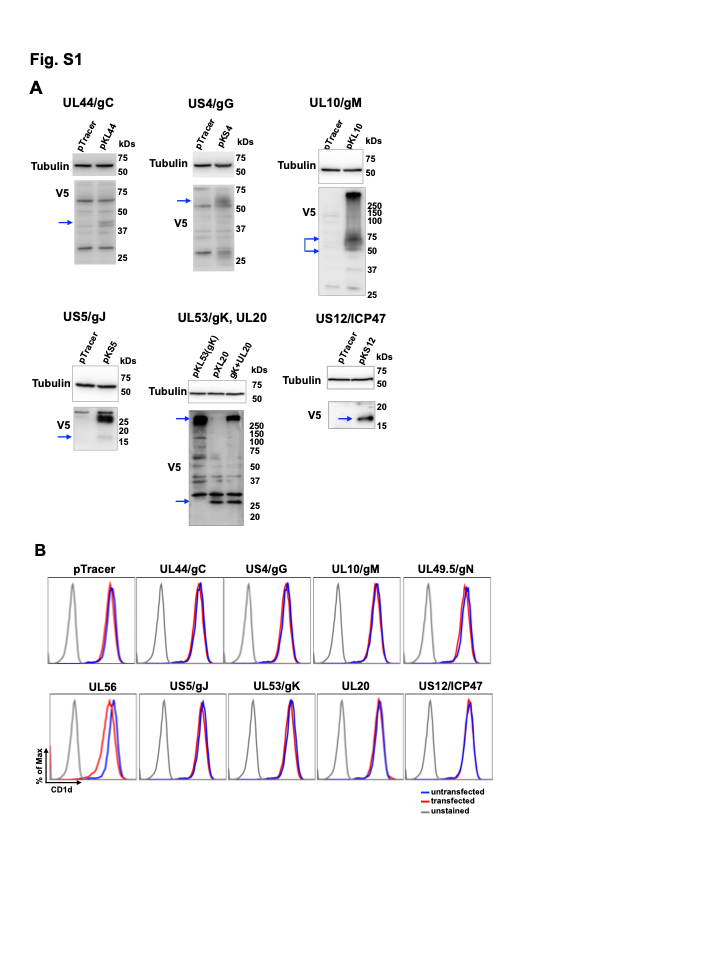

Supplement: Figure S1 — Examination of potential CD1d downregulation by HSV-1 glycoproteins and ICP47. [file jvi.02140-24-s0001.tiff]

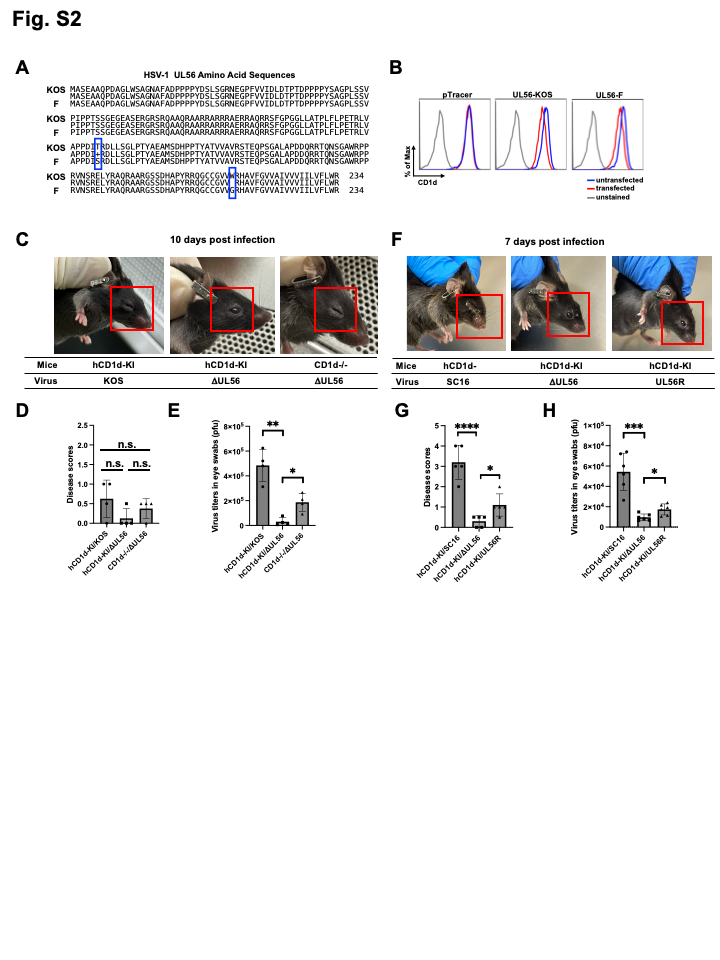

Supplement: Figure S2 — CD1d downregulation and evasion of NKT cell function is conserved in HSV-1 KOS and F strains. [file jvi.02140-24-s0002.tiff]

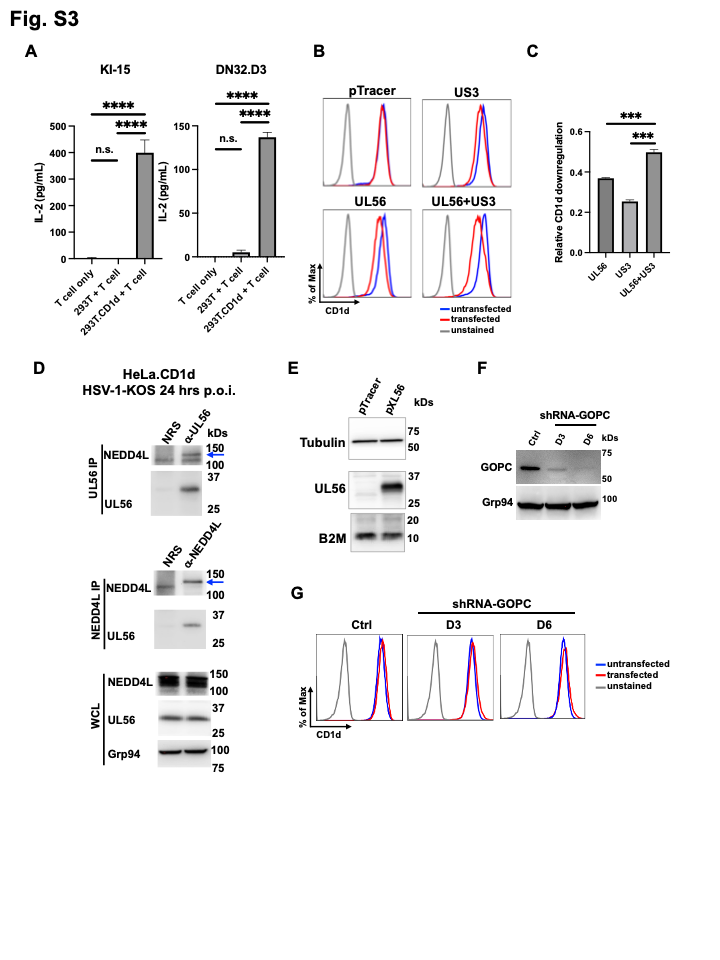

Supplement: Figure S3 — Specificity and mechanism of HSV-1 UL56 downregulation of CD1d expression. [file jvi.02140-24-s0003.tiff]
