# Supplementary material for: HSV-1 UL56 protein recruits cellular NEDD4-family ubiquitin ligases to suppress CD1d expression and NKT cell function
Source: J Virol. 2025 Mar 6;99(4):e02140-24. doi: 10.1128/jvi.02140-24 (PMC11998485; doi:10.1128/jvi.02140-24)
Supplement: Supplemental legends — Legends for Fig. S1 to S3. [file jvi.02140-24-s0004.pdf]

Figure legends for supplemental figures:

Fig. S1. Examination of potential CD1d downregulation by HSV-1 glycoproteins and ICP47. (A). Verification of expression of HSV-1 glycoproteins. 293T.CD1d cells were transfected with pTracer alone or co-transfected with pTracer and plasmids constructs expressing various glycoproteins (all V5-tagged) and blotted with anti-V5 antibodies. Arrows pointed to detected viral glycoproteins or ICP47 protein. (B). Transfected 293T.CD1d cells were stained for cell surface CD1d expression and analyzed by flow cytometry. Transfected cells were gated by GFP fluorescence.

Fig. S2. CD1d downregulation and evasion of NKT cell function is conserved in HSV-1 KOS and F strains. (A). Alignment of UL56 amino acid sequences between HSV-1 KOS and F strains. The two different amino acids were boxed. (B). 293T.CD1d cells were transfected with pTracer alone or co-transfected with pTracer and plasmids constructs expressing UL56-KOS or UL56-F proteins. Cells were stained for surface CD1d expression and analyzed by flow cytometry. (C-E). hCD1d-KI or CD1d-/- mice were ocularly infected with wild-type (KOS) or UL56-deficient (ΔUL56) HSV-1 KOS strain. (C). Representative mouse eye images at ten days post infection. (D). Disease scores from infected mice ten days post infection were plotted. (E). Eye swabs from infected mice two days post infection were titered by plaque assays. (F-H). hCD1d-KI mice were ocularly infected with wild-type (SC16), UL56-deficient (ΔUL56), and UL56-repaired HSV-1 SC16 strain. (F). Representative mouse eye images at seven days post infection. (G). Disease scores from infected mice seven days post infection were plotted. (H). Virus titers from mouse eye swabs one day post infection were plotted. Statistical analysis by One-way ANOVA with Duncan post hoc multiple comparisons. n.s.: not significant. *: $p<0.05$, **: $p<0.01$, ***: $p<0.001$, ****: $p<0.0001$.

Fig. S3. Specificity and mechanism of HSV-1 UL56 downregulation of CD1d expression. (A, B). Collaboration of HSV-1 US3 and UL56 proteins in CD1d downregulation. (A). 293T.CD1d cells were transfected with pTracer alone or co-transfected with pTracer and plasmids constructs expressing US3, UL56 or both proteins. Cells were stained for surface CD1d expression and analyzed by flow cytometry. (B). Statistical analysis of results from replica experiments by Student's T test or One-way ANOVA with Duncan post hoc multiple comparisons. n.s.: not significant, ***: $p<0.001$. (C). Specificity of CD1d antigen presentation in 293T.CD1d cells. 293T or 293T.CD1d cells were loaded with α-GalCer and co-cultured with either KI-15 or DN32.D3 iNKT cell hybridoma cells. Cell culture supernatants were subjected to mouse IL-2 ELISA for analysis of iNKT cell activation. (D). UL56 interacts with NEDD4L in HSV-1-infected cells. HeLa.CD1d cells were infected by HSV-1 KOS strain for 24 hours. Cells were lysed and subjected to immunoprecipitation with antibodies against either UL56 or NEDD4L proteins. The whole cell lysates or immunoprecipitants were analyzed by SDS-PAGE and blotted with indicated antibodies. Arrows pointed to detected NEDD4L proteins. (E). B2M is not downregulated by UL56 protein expression. 293T.CD1d cells were transfected with either pTracer or plasmid expressing HSV-1 UL56 (pXL56) and cell lysates were subjected to western blotting with indicated antibodies. (F, G). GOPC knockdown does not affect CD1d expression. (F). 293T.CD1d cells were transfected with pTracer and plasmids expressing non-specific control (Ctrl), D3 or D6 shRNAs targeting human GOPC gene. GOPC protein levels in cell lysates were analyzed by western blotting with Grp94 as control protein. (G). Cell surface CD1d expression in transfected 293T.CD1d cells was analyzed by flow cytometry using GFP fluorescence for gating of transfected cells. Statistical analysis by One-way ANOVA with Duncan post hoc multiple comparisons. n.s.: not significant. *: $p<0.05$, **: $p<0.01$, ***: $P<0.001$, ****: $P<0.0001$.
